# Internet Risks: An Overview of Victimization in Cyberbullying, Cyber Dating Abuse, Sexting, Online Grooming and Problematic Internet Use

**DOI:** 10.3390/ijerph15112471

**Published:** 2018-11-05

**Authors:** Juan M. Machimbarrena, Esther Calvete, Liria Fernández-González, Aitor Álvarez-Bardón, Lourdes Álvarez-Fernández, Joaquín González-Cabrera

**Affiliations:** 1 Faculty of Education, International University of la Rioja (UNIR), Avenida de la Paz, 137, 26006 Logroño, La Rioja, Spain; juan.machimbarrena@unir.net (J.M.M.); aitor.alvarez@unir.net (A.Á.-B.); marialourdes.alvarez@unir.net (L.Á.-F.); 2 Faculty of Psychology and Education, University of Deusto. Unibertsitate Etorb., 24, 48007 Bilbao, Bizkaia, Spain; esther.calvete@deusto.es (E.C.); liria.fernandez@deusto.es (L.F.-G.)

**Keywords:** cyberbullying, cyber dating abuse, grooming, sexting, problematic Internet use, adolescence, Internet risks, prevalence, polyvictimization

## Abstract

The advance of digital media has created risks that affect the bio-psycho-social well-being of adolescents. Some of these risks are cyberbullying, cyber dating abuse, sexting, online grooming and problematic Internet use. These risks have been studied individually or through associations of some of them but they have not been explored conjointly. The main objective is to determine the comorbidity between the described Internet risks and to identify the profiles of victimized adolescents. An analytical and cross-sectional study with 3212 participants (46.3% males) from 22 Spanish schools was carried out. Mean age was 13.92 ± 1.44 years (range 11–21). Assessment tools with adequate standards of reliability and validity were used. The main results indicate that the most prevalent single risk is cyberbullying victimization (30.27%). The most prevalent two-risk associations are cyberbullying-online grooming (12.61%) and cyberbullying-sexting (5.79%). The three-risk combination of cyberbullying-sexting-grooming (7.12%) is highlighted, while 5.49% of the adolescents present all the risks. In addition, four profiles are distinguished, with the profile Sexualized risk behaviour standing out, with high scores in grooming and sexting and low scores in the rest of the risks. Determining the comorbidity of risks is useful for clinical and educational interventions, as it can provide information about additional risks.

## 1. Introduction

The digital society is an opportunity for personal development in many fields related to social, health, educational and economic aspects. Although digital media provides great advantages (such as rapid communication, information availability, opportunities for learning and entertainment), its use is not without potential risks. According to the co-construction model [1], through digital media, adolescents construct and co-construct their environments, connecting their online and offline worlds. Therefore, digital worlds may serve as a playground for important developmental tasks of adolescence such as sexuality and identity but also, adolescents have to deal with the darker and more unsavoury aspects of technology [2], during a stage of special psychological vulnerability [3]. Given that digital media presents some unique challenges for sexuality, intimacy, aggressive behaviour and problematic use that affect boys and girls and older and younger youth in different ways [2,3], participants’ details (such as their sex and age) are an important issue when studying online behaviour.

High-risk situations are mainly due to the recent substantial increase in the use of Internet. This can be confirmed through the survey published in 2015 by the Pew Research Centre, which reported that 24% of youngsters between 13 and 17 years are constantly connected to Internet and 56% of them several times a day [4]. In this same line, the results of the CIBERASTUR (Project about bullying, cyberbullying, problematic uses of internet and quality of life related to health between 10 and 18 years of the Principality of Asturias) study performed in Spain with more than 25,000 adolescents aged 11–18 years showed that 95.7% reported owning a smartphone and, among them, up to 86.6% used it daily. Regarding the time of connection, 20.6% of the adolescents reported spending five hours or more from Monday to Friday and on weekends, this increased to 33.2% [5].

The increasing possession of smartphones, as well as their greater everyday use, is the gateway to potential risks that negatively affect bio-psycho-social well-being [6]. In the current literature, there are many approaches under the heading of Internet risks. The authors of this study define these risks as a set of psychosocial problems that are characteristic of Internet, initiated and maintained in an online context that has a mutual and bidirectional relationship with the individual’s off-line reality. These risks can have severe outcomes for victims, who often present internalizing and externalizing problems [7,8,9,10], loss of perceived quality of life [11], suicidal ideation [9,12] and interference in academic, social and family life [13].

Moreover, although most of these risks have been assessed, particularly cyberbullying, the interconnections among them have been neglected. However, research has shown that victimization in one context can make youth vulnerable to other types of victimization and thus extend their victim status over time. According to the polyvictimization theory [14], victimization often does not occur in isolation but is frequently followed by other forms of abuse. These authors highlighted the importance of identifying children and adolescents who had experienced multiple victimizations because they could develop more severe psychological problems. In this sense, research shows that populations involved in multiple risk behaviours have the greatest risk for chronic diseases, psychiatric disorders, suicidal behaviours and premature death compared to individuals with single or no risk behaviours [15]. This also falls in line with several theories, such as the cumulative risk model [16], which states that most children experiencing a single psychosocial risk factor might suffer little or no enduring harm, whereas a subset of children experiencing multiple risk factors are much more likely to experience psychological disorders [17].

In line with the above-mentioned theories, this study aims to explore five possible adolescent risks in the digital media (cyberbullying victimization, cyber dating abuse victimization, sexting, online grooming and problematic Internet use), considering their interconnections and comorbidities.

Cyberbullying is a violent and intentional act that is performed repeatedly, over a long period of time, through the use of the new technologies, by one or more persons directed against another person who has difficulties to defend him- or herself. In addition, it is usually anonymous and can occur at any time and place [18]. To form a general idea of the current situation, a review of 159 studies determined that the prevalence of cyberbullying last year ranged between 1% and 61.1% [19]. In this sense, a study on cyberbullying in all the Spanish regions showed average victimization values near 25% [20]. The prevalence of victimization tends to be higher in girls than in boys [21,22,23], although some reviews indicate that the results are mixed and there is no unanimity [24,25]. Regarding differences based on age, cyberbullying appears to increase as children approach adolescence, reaching its maximum prevalence around age 15 [18].

In comparison with cyberbullying, cyber dating abuse has received less attention [26,27]. This phenomenon comprises a wide range of behaviours that include, for example, attempts to control one’s partner or ex-partner via digital media [28] and/or by sending insulting or threatening messages [29]. Regarding victimization in Spain, rates of 75% and 14%, respectively, for controlling behaviour and insulting or threatening messages, have been obtained [30]. In terms of sex differences, although girls report higher sexual victimization than boys between ages 12–18 [29], the results depend on the type of behaviour analysed and are disparate in age ranges above 18 years [28,30,31].

Online grooming is a serious social problem and it is often considered a criminal offence, like in Spain, where it is included in the Criminal Code [32]. Grooming has been defined as the process by which an adult, using digital media prepares a minor in order to obtain sexual material (images, videos) from him or her or to sexually abuse him or her [33]. Studies of surveys of adolescents aged 10–17 indicate a prevalence of sexual requests ranging between 5% and 9% [34]. Prevalence figures of 15.6% for girls and 9.3% for boys have been found in a Spanish population study of young people aged 12 to 15 years [35].

Sexting refers to the act of sending a peer photographs and videos with some level of sexual content, taken or recorded by the protagonist, through the use of digital media [36]. In the international context, the prevalence data on sexting vary between 9.6% and 54% [37,38]. In Spain, the few works carried out found a sexting prevalence rate of 13.5% [39]. Although some studies suggest that sexting is practiced more by girls [40], other studies have revealed that there seems to be no differences between boys and girls, with prevalence rising as age increases [41].

Finally, problematic Internet use stresses the possible dysfunctions that Internet consumption can imply in the person’s life [42]. Preference for online social interaction and mood regulation through Internet increase the likelihood of presenting poor self-regulation, which has several negative consequences in the person’s life [43]. In a recent study in Spain, 4% of the students presented problematic use or a pattern of risk and nearly 40% had presented some occasional problematic use in the last seven months [5]. The epidemiological studies report prevalence rates that reveal the clinical and social relevance of this problem [44], which, moreover, is increasing with age [45]. In relation to sex differences, greater problematic Internet use has been observed in females [5,45,46] although other studies find no differences [21].

As mentioned, the available research has generally focused on a single risk or has only addressed the associations between some of these phenomena. Thus, recent studies have linked cyberbullying with problematic Internet use [21], cyber dating abuse victimization with problematic Internet use and sexting [26] and cyber dating abuse victimization and perpetration with cyberbullying victimization and aggression [29], sexting with cyber dating abuse perpetration and victimization [31,47] and grooming with sexting and cyberbullying victimization [35]. However, to our knowledge, the comorbidity of this series of risks has not been evaluated in order to establish possible typologies of risks. The development of typologies that integrate several modalities of Internet victimization could contribute to extend the polyvictimization theory [9,14].

Therefore, the main objective of this work is to determine the comorbidity between Internet risks (cyberbullying victimization, cyber dating abuse victimization, sexting, online grooming and problematic Internet use) and to identify the profiles of adolescents as a function of the presence of these risks. The secondary goals are to provide the latest data on the prevalence of victimization due to Internet risks and to analyse differences according to sex, educational stage and type of school (private or public centre).

We expect to find high comorbidity among the risks and to observe the emergence of several distinct profiles. Regarding the secondary objectives, considering the results of previous studies, our working hypothesis is that cyberbullying victimization will be the Internet risk with the highest prevalence [19]. Like in diverse studies, we expect to find differences between boys and girls, with higher victimization scores in girls [5,20,21,22,23,29,40,45,46]. We also expect that the higher the educational stage, the higher will be the scores in the diverse risks, given that age facilitates access to Internet [18,41,45,48].

## 2. Materials and Methods

### 2.1. Design and Participants

An analytical and cross-sectional study was performed between December 2017 and April 2018. The initial sample comprised 3286 participants. After the elimination of some participants because of the short time they spent completing the battery of questionnaires (less than 10 min) (*n* = 69) or extreme ceiling effects of their scores (scoring the maximum scores in all measurements) (*n* = 5), the final sample was made up of 3212 participants. Forty-six point three percent were boys (*n* = 1487) and 53.7% were girls (*n* = 1725). The mean and standard deviation of age was 13.92 ± 1.44, with a range of 11–21 years. Concerning educational level, 53.4% (*n* = 1714) of the sample were in 1st–2nd grade of Compulsory Secondary Education (CSE) (11–13 years approximately), 40.7% (*n* = 1307) were in 3rd–4th grade (14–16 years approximately) and 5.9% (*n* = 191) were studying Post-secondary Education (16–21 years). Sampling procedure was non-probabilistic and incidental but the sample included participants of 122 classrooms from 22 schools in 7 regions of Spain (Basque country, Asturias, Castilla-Leon, Castilla la Mancha, Valencia, Aragón and Madrid) took part in the sample collection. Sixteen schools were private (*n* = 2554) and six were public (*n* = 658).

### 2.2. Instruments

The participants provided information about demographic variables such as sex, grade, school and age. The following instruments were used to analyse the variables under study, always referring to the past five months.

Victimization Scale of the Cyberbullying Questionnaire (CBQ) [49,50]. This consists of 9 items about victimization by cyberbullying behaviours (e.g., “Sending me threatening or insulting messages”). For this study, we adapted the response format of the items to a 5-point Likert scale ranging from 0 (*never*) to 4 (*almost every week*). Reliability for this study was: 0.81 for the Cronbach alpha, 0.87 for the ordinal alpha and 0.87 for omega.

Victimization Scale adapted from the Cyber Dating Abuse Questionnaire [30]. This consists of 11 items referring to different types of cyber dating abuse, including behaviours of controlling the partner’s mobile and insulting (e.g., “called me or chatted excessively with me to control where I was and with whom”). A four-point response scale was employed ranging from 0 (*never*) to 3 (*almost always*). Only participants who reported having had a partner during the last six months completed this questionnaire. The Cronbach alpha obtained was 0.87, the ordinal alpha was 0.91 and the omega was 0.91.

The sexting questionnaire adapted from Gámez-Guadix et al. [51]. Three items about sending photos, information or videos of sexual or intimate content to three potential recipients: a partner, a friend and someone they have met on Internet but not in person (e.g., “Have sent information, photos or videos with intimate or sexual content about yourself”). A five-point response scale was used ranging from 0 (*never*) to 4 (*7 or more times*). The reliability obtained for this study was 0.70 for the Cronbach alpha, 0.85 for the ordinal alpha and 0.85 for the omega.

Questionnaire for Online Sexual Solicitation and Interaction of Minors with Adults [52]. It contains 11 items to evaluate the sexual interactions that are part of the initiation, process, or result of online grooming (e.g., “An adult has asked me to have cybersex”). Items are rated on a four-point response scale ranging from 0 (*never*) to 3 (*6 or more times*). Reliability for this study was 0.89 for the Cronbach alpha, 0.91 for the ordinal alpha and 0.92 for the omega.

Spanish version of the Generalized and Problematic Internet Use Scale (GPIUS2) [43]. It presents 15 items referring to various aspects of problematic Internet use such as Preference for online social interaction, Poor self-regulation and Negative consequences (e.g., “I think obsessively about connecting when I’m not connected”). Agreement with the items is rated on a six-point Likert scale ranging from 0 (*completely disagree*) to 5 (*completely agree*). Reliability for this study was 0.91 for the Cronbach alpha, 0.92 for the ordinal alpha and 0.92 for the omega.

To standardize the different Internet risks, a combination of statistical and criteria standards was followed. The general statistical standards for cyberbullying victimization, cyber dating abuse victimization, sexting and problematic Internet use are: (i) No Problem (a total score of 0 or 1 [in the case of sexting, only a total score equal to 0]); (ii) Occasional Problems (a score below one standard deviation); (iii) Moderate Problems (scores between one and two standard deviations); and (iv) Severe Problems (scores equal to or above two standard deviations). For the cyberbullying criteria, we also considered a single behaviour reported almost every week as a severe problem. Finally, for online grooming, due to its perniciousness and reflection in the penal code, it was considered a problem when a participant scored 1 or more, there being only two categories (No Problem and Problem).

### 2.3. Procedure

The battery of questionnaires was applied in online format through Qualtrics. Participants completed the questionnaires in the different computer classrooms or through tablets coordinated by the orientation departments of each school and under the supervision of the classroom tutor. The questionnaires were completed during regular school time. It was stressed that the students should answer truthfully and should not pause for a long time at any particular question. The time needed to fill out the questionnaires ranged mostly between 20 and 35 min (mean time = 24 min, SD = 4 min), depending on students’ age and reading comprehension. A procedure to provide information and obtain guardians’ passive consent was established. Participating students’ and families’ collaboration was voluntary, anonymous and disinterested. To ensure the privacy of their answers, participants did not indicate their name or any identifying data. Questionnaires were completed on different computers on an individual basis and responses were automatically saved on the server. In this way, no information of the questionnaire remained in the computers used. To implement the project, a formal request was presented to the Research Ethics Committee of the Principality of Asturias (Ref. 231/17) and to the Ethics Committee of the University of Deusto.

### 2.4. Statistical Analysis

Statistical analyses were carried out using the Statistical Package for the Social Sciences (SPSS) [53], R software [54] and the tidyLPA package [55]. Firstly, to determine the internal consistency of the instruments, Cronbach alphas [56], ordinal alphas [57] and omega coefficients [58] were estimated. Next, we confirmed the assumptions of normality (Shapiro-Wilks’ statistic) of the target variables of the study, as well as the homogeneity of variances to compare the groups (Levene’s test).

In order to explore the comorbidity between the different Internet risks, firstly, we calculated the standardized scores of the variables for which relations had been established and then, we calculated Pearson correlations. Next, to identify adolescent profiles according to the presence of these risks, we performed a Latent Profile Analysis (LPA). For this purpose, exploratorily, we compared different solutions according to the Bayesian Criterion Information (BIC) and the Aikake Information Criterion (AIC). The model with the best fit was the one with equal variances and covariances fixed to 0 (class-invariant parameterization). After identifying the number and nature of the profiles, the participants were assigned to their most likely profile based on their posterior probabilities.

Finally, regarding the secondary goals of the study of providing current data on the prevalence of victimization due to Internet risks and analysing the differences as a function of sex, educational stage and type of school, the following analyses were conducted: (i) analysis of frequencies and central tendency and dispersion measures of the target variables; (ii) chi-square analysis to contrast the proportions and analysis of the adjusted standardized residuals; (iii) Student’s *t* for dependent and independent samples (or failing that, Welch’s *t*-test); and (iv) in those cases where statistically significant differences were found, Cohen’s *d* was calculated; (v) analysis of variance with Games-Howell post-hoc comparisons. A value of less than *p* = 0.05 was considered significant.

## 3. Results

Table 1 depicts the prevalence of each of the risks of the study, depending on the degree of severity found. Additionally, it shows the comparisons between the distributions of boys and girls for the different risks. Overall, the participants who showed no problem ranged between 46.25% who had no problems with problematic Internet use and up to 83.4% who had no problems with online grooming. We note that the range of moderate and severe problems varied between 4% for sexting and 17% for problematic Internet use. In cyber dating abuse, there was up to 10.9% of moderate/severe problems and in cyberbullying, it reached 13.7%. The frequencies found in the different levels of problems were usually greater for girls than for boys.

In this regard, significant differences were also found between boys and girls in the mean total scores of cyberbullying victimization (Welch’s *t* = −2.02, *p* < 0.043, *d* = 0.07), online grooming (Welch’s *t* = −3.51, *p* < 0.001, *d* = 0.12) and problematic Internet use (Welch’s *t* = −2.07, *p* < 0.039, *d* = 0.07). In these cases, the mean scores were higher for girls than for boys. There were no significant differences in the rest of the risks: cyber dating abuse victimization (Welch’s *t* = −1.9, *p* < 0.058, *d* = 0.12) and sexting (Welch’s *t* = 0.94, *p* < 0.410, *d* = 0.03).

Regarding the type of school (private and public), significant differences were only found in the risks of online grooming (*t* = −3.37, *p* < 0.001, *d* = 0.13) and sexting (*t* = 3.8, *p* < 0.001, *d* = 0.15). The mean scores were higher in public schools than in private schools in both cases.

In terms of the educational stage (1st–2nd grade of CSE, 3rd–4th grade of CSE and Post-secondary Education), statistically significant differences were found for the risks of cyberbullying victimization (*p* < 0.002), online grooming (*p* < 0.001), sexting (*p* < 0.001) and problematic Internet use (*p* < 0.001). The scores were higher in 3rd–4th grades, except for online grooming victimization, where higher scores were found in Post-secondary Education (see Table 2).

Table 3 shows the correlations between the various risks. All of them had positive and significant correlations with each other, with the relationship between cyberbullying victimization and cyber dating victimization standing out. Internet risks with a sexual component (online grooming and sexting) were highly correlated. In general, the correlations were higher for boys in most of the risks, with the exception of the relationships between cyber dating victimization and grooming and between problematic Internet use and cyberbullying victimization, online grooming and sexting.

Table 4 presents the comorbidities among the various Internet risks related to personal interaction (cyberbullying victimization, cyber dating abuse victimization, sexting and online grooming). Only the participants who completed all the items concerning risks related to victimization (*n* = 1109) were considered (i.e., eliminating from the study those who had no partner). Of the remaining participants, 60.7% presented at least one of the analysed risks (*n* = 674). The risk with the highest individual prevalence was cyberbullying victimization (30.27%), followed by online grooming. The most prevalent two-risk combinations were cyberbullying victimization-online grooming and cyberbullying-sexting. We highlight the three-risk combination of cyberbullying-sexting-grooming victimization. Finally, 5.49% of the victimized adolescents presented all the risks conjointly.

As a continuation of the above, to delve into the different victim profiles, a Latent Profile Analysis (LPA) was performed. For this purpose, the scores on the variables of victimization by cyberbullying, cyber dating abuse, sexting, online grooming and problematic Internet use were considered. Table 5 depicts the values for the different models, with the four-profile model being the most appropriate for our research, as it showed the best fit and high entropy.

**Figure 1:**
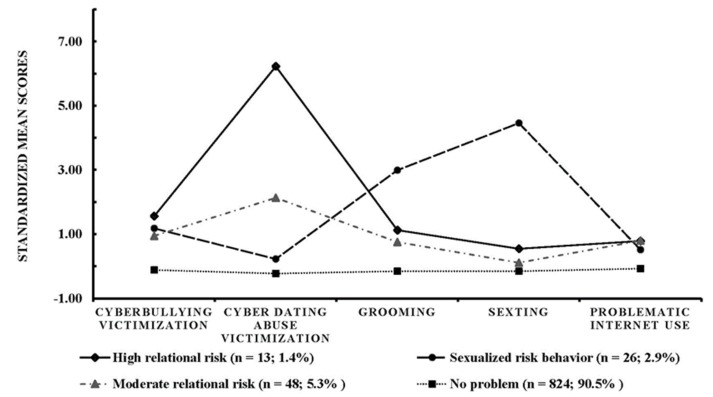
Classes resulting from the latent profile analysis (standardized scores).

Figure 1 presents the four-profile model, where a clear “no problem” profile was distinguished. This was characterized by scores lower than the mean in all the variables of the study. The rest of the profiles presented high comorbidity with each other, with similar levels of cyberbullying victimization and problematic Internet use. However, we note the so-called “High relational risk” profile due to its high scores in cyberbullying victimization and cyber dating abuse victimization. The “Moderate relational risk” profile pointed in this same direction, showing a similar pattern to the previous profile but with a more moderate tendency in the set of risks. Lastly, the “Sexualized risk behaviour” profile presented the lowest scores of all three risk profiles in cyber dating abuse victimization but some very high scores in grooming and sexting victimization. Regarding the distribution of the profiles by sex and age, the contingency analysis of sex and profile indicated a significant relationship between the two variables, χ^2^(3, *N* = 911) = 15.44, *p* = 0.001, *V* = 0.13. Analysis of the adjusted standardized residuals indicated a higher number of girls than expected in the “Sexualized risk Behaviour” profile (1.4% [*n* = 6] of boys and 4.2% [*n* = 20] of girls; standardized residual = 2.5) and in the “Moderate relational risk” profile (3% [*n* = 13] of boys and 7.3% [*n* = 35] of girls; standardized residual = 2.9) and a higher frequency of boys with “no problem” (94% [*n* = 407] of boys and 87.1% [*n* = 417] of girls; standardized residual = 3.7). As for the grade, the chi square analysis was nonsignificant, χ^2^(6, *N* = 911) = 10.11, *p* = 0.120, *V* = 0.07. However, the analysis of standardized residuals revealed a higher frequency than expected in Post-secondary Education students in the “High relational risk” profile (1.2% [*n* = 5] of students of 1st–2nd grade of CSE, 1.0% [*n* = 4] of students of 3rd–4th grade of CSE and 4.8% [*n* = 4] of Post-secondary Education students, standardized residual = 2.7).

## 4. Discussion

The main goal of this study was to determine the comorbidity of Internet risks and to identify adolescents’ profiles according to the presence of these risks. Results show that up to 24.5% of the victimized adolescents presented two simultaneous risks, 17.4% presented three risks and 5.4% had all the analysed risks. The most common risk is cyberbullying victimization, both when it occurs by itself (in 30.3%), or when it does so in conjunction with other risks, thereby amounting to 75% of the cases. Some particularly relevant combinations are: cyberbullying-grooming victimization, with 12.6% of the students, or cyberbullying-sexting-grooming victimization, with 7.1%. These results point in the same direction as other studies that found the relationship between several of the studied Internet risks [26,29,31,35,47] but it extends the results of these studies, adopting a more comprehensive perspective that considers multiple Internet risks. These data related to the model of co-construction [1,2], which posits that adolescents use digital media in such a way that their online and offline worlds are joined in a single reality; therefore, these risks pose a major problem for their development. In addition, they show a clear relationship with the theories of polyvictimization [9,14] and cumulative risk model [16,17], revealing that victimization often does not occur in isolation.

Further to the above, analysis of the profiles revealed four distinct groups, showing a profile with greater sexual salience (related to high scores in grooming and sexting) and two of them related to the risk of cyber dating abuse victimization, depending on their intensity (moderate or severe). Finally, there was a profile that grouped the set of participants with no problems or with mild problems. This work makes a singular contribution and there are no previous studies that compare the results obtained. However, some implications of the profiles obtained can be derived. Firstly, there is evidence of a group of adolescents who are particularly vulnerable to sexual victimization. As mentioned, whereas grooming is a serious problem, often constituting a criminal offense, sexting in itself does not necessarily constitute a harmful practice. In this sense, it is suggested that preventive interventions should focus on making adolescents aware of its potential dangers, especially when practiced irresponsibly. According to the findings of previous studies, 10% of adolescents have sometimes sent material with sexual or intimate content to people whom they had met on Internet but not in person [31]. This result is consistent with the profile of sexual risk that emerged in the present study, which shows a relationship between sexting and grooming. On the other hand, another profile was found of adolescents who predominantly show a risk associated with cyber dating abuse victimization, suggesting that there are differential factors that increase the likelihood of becoming a victim of cyber dating abuse (and not necessarily of other kinds of cybervictimization).

Regarding the secondary objectives, we analysed the prevalence of the different risks. As hypothesized, the most prevalent risk was cyberbullying victimization, which was suffered in any of its forms (occasional, moderate, or severe) by 33.1% of the total sample. This coincides with the prevalence data of other studies [19,20,21]. Similarly, the percentage of participants presenting problems of cyber dating abuse victimization (17.4%) is consistent with other studies of the same context that found a prevalence of 14% [30]. The results in terms of prevalence of other risks present some differences with other studies. This is the case of sexting, where a prevalence of 9.5% was obtained, which is slightly lower than other studies, which placed it around 14% [41,52]. This may be due to the strict criteria of standardization used in this work, or with the differences in the samples (adolescents vs. adults). At the opposite pole, the results of the prevalence of online grooming and problematic Internet use were higher than those of several studies. In the case of grooming, the results indicate 16.6% of victims, a higher number than found in other Spanish and international studies indicating 5–9% [34] and 9–15% [35]. In the case of problematic Internet use, 53.5% of the participants presented at least occasional problematic Internet use, which is higher than other Spanish studies that estimated problematic Internet use at 40% [5]. This may be due to the increase of this problem among adolescents [45].

Regarding differences according to sex, as hypothesized, we found that there is generally a higher percentage of victimized girls in the different risks. In the same vein, girls presented higher mean scores in cyberbullying victimization and online grooming and in problematic Internet use. This is consistent with studies that indicate similar results in cyberbullying [21,22,23], sexting [40], grooming [35] and problematic Internet use [5,45,46] and adds to the debate with those studies and reviews that found no differences in cyberbullying victimization [24,25], sexting [41] and problematic Internet use [21]. This is an important finding not only for future research that should analyse further differences in use of digital media and victimization and their underlying cause but also for future prevention and intervention programs that should take these differences into account to be more effective. Regarding differences according to age, we observed, in general, that 1st–2nd graders obtained lower scores, followed by 3rd–4th graders of CSE. As hypothesized, this suggests that involvement in these problems increases with age, which also tends to be related to having a smartphone and to its greater use [41,45,46]. These findings are consistent with those obtained in other studies of cyberbullying [18,21,59,60], sexting [41] and problematic Internet use [5,45,46] and point to older adolescents as a vulnerable group. This is of the utmost important for the future design of prevention programs that should be aimed at younger adolescents in order to prevent some of these risks that appear to be more prevalent during last years of secondary and post-secondary education. Finally, differences depending on the type of school showed higher scores in online grooming and sexting in public schools, although the effect size was small and there are no prior studies that have assessed these risks in relation to the type of school.

As previously mentioned, it is important to make it explicit that, on an individual basis, many of these risks have been associated with serious problems. For instance, cyberbullying is related to the loss of perceived quality of life [11], suicidal ideation [12], depressive mood [7] and physiological impact on the hypothalamic-pituitary-adrenal axis (HPA) [61]. Other research has shown the relationship between cyber dating abuse victimization and depression and anxiety [8]. Sexting has also been significantly associated with symptoms of depression, impulsivity and substance use [10]. Online grooming presents a complex psychological impact that is associated with anxiety, stress, depression, phobias, low self-esteem, feelings of guilt and shame, as well as suicidal ideation and self-inflicted injuries [9]. Finally, problematic Internet use has also been linked to many problems such as loss of quality of life, changes in healthy habits (sleep, eating, physical activity, etc.) and interference in the family, social and academic life [13]. These links with depression are of the utmost importance, not only because of their relationship with other adaptive and clinical variables from an early age [62] but also because unipolar and bipolar disorders present impairments in the white and grey matter compartments [63]. Therefore, if one or two risks already encompass multiple problems for adolescence [29,31,47], the possible comorbidity of three or more could generate even worse effects among those who suffer from them, as suggested by the theories of polyvictimization [9,14] and of the cumulative risk model [16,17].

The study has several practical implications at the clinical level (with units of paediatrics, psychology and psychiatry) and at the educational level (with tutors, counsellors and school psychologists, among others). The data show the overlap between the different risks and allow us to weigh possible assessments when detecting one of them (especially cyberbullying). In any case, the existence of any of the risks should be a warning to professionals of the possible simultaneous emergence of others in order to promote their prevention. Because of the comorbidity data obtained, the need for further research is suggested to study the factors of vulnerability and protection potentially shared by all the Internet risks described. In addition, the existing strategies and prevention programs for some of the risks (mainly cyberbullying) should be re-assessed to determine whether they have an impact on some of the additional risks studied [64,65]. The existing programs may have a positive impact but if this were not the case, their modification or the creation of new programs should be considered, which would use a new inclusive approach towards the diverse risks, with special emphasis on the child’s protection and the responsible use of Internet. In this sense, the sex and age differences found are of particular relevance, as they seem to indicate the need for prevention in the early stages of secondary education, before the problem reaches higher prevalence rates. In addition, our results indicate that girls are more vulnerable; the reasons for these differences require further research but they are important when it comes to planning and implementing prevention programs. To close this point of implications, we also suggest that future research should consider the joint view of Internet risks, because adolescents are currently exposed to multiple forms of cybervictimization and this may continue to increase in the future.

This study has some limitations. First, the results are based on self-reports with the entailed response bias. To alleviate this limitation, we propose that future research use additional measures such as sociograms or reports of parents/teachers/peers. In relation to the assessment tools, we used adaptations to Spanish of some versions (such as problematic Internet use) or other nationwide instruments of reference within the context of our research. However, we recommend caution when comparing prevalence results with other studies and other measuring instruments, as there is considerable heterogeneity between constructs, instruments and ways to establish criteria. Furthermore, the sampling procedure was non-probabilistic and incidental. Although the sample was large and included more than 122 classrooms distributed in seven different Spanish regions, it may be not statistically representative of the Spanish adolescent population. In addition, the nature of the risk of cyber dating abuse victimization, for which participants had to indicate having a partner, led to a reduction in the number of participants in some analyses (comorbidity and LPA). Finally, this study addresses several of the most important Internet risks. However, other risks such as nomophobia, the Fear of Missing Out (FoMO), or Internet Gaming Disorder (IGD) were not included. In this sense, we propose future research to include these or other risks and to search for greater convergence in the assessment tools and in the definition of these risks, as well as to use a longitudinal design that allows analysing the stability of the profiles over time.

## 5. Conclusions

The findings of this study suggest the existence of comorbidities among different Internet risks, with cyberbullying victimization being the most prevalent single risk. In addition, four profiles of the described Internet risks were observed, a well-adjusted one (that scores lower than the mean in all the Internet risks), one related to sexual behaviours (sexting and online grooming) and another two related to cyber dating abuse victimization. In the most practical sense, the study suggests that educational professionals should appraise possible assessment of other risks when a person presents cybervictimization.

## Figures and Tables

**Table 1:** Prevalence of each of the risks as a function of the severity of the problem for the total sample and of sex.

Construct	Severity of Problem	Total *f* (%)	Boys *f* (%)	Girls *f* (%)	χ^2^ (*p*)
Cyberbullying victimization	No problem	2052 (66.9)	988 (48.1) *	1064 (51.9) **	7.72 (0.052)
	Occasional	595(19.4)	253 (42.5) **	342 (57.5) *	
	Moderate	200 (6.5)	68 (44.0)	90 (56.0)	
	Severe	220 (7.2)	114 (43.5)	148 (56.5)	
Cyber dating abuse victimization	No problem	876 (82.6)	442 (50.5) *	434 (49.5) **	10.01 (0.019)
	Occasional	69 (6.5)	25 (36.2) **	44 (63.8) *	
	Moderate	62 (5.8)	23 (37.1)	39 (62.9)	
	Severe	54 (5.1)	22 (40.7)	32 (59.3)	
Grooming	No	2610 (83.4)	1278 (49.0) *	1332 (51.0) **	40.26 (0.000)
	Yes	521 (16.6)	176 (33.8) **	345 (66.2) *	
Sexting	No problem	2823 (90.5)	1306 (46.3)	1517 (53.7)	0.81 (0.994)
	Occasional	173 (5.5)	79 (45.7)	94 (54.3)	
	Moderate	31 (1.0)	15 (48.4)	16 (51.6)	
	Severe	93 (3.0)	43 (46.2)	50 (53.8)	
Problematic Internet use	No problem	1316 (46.5)	625 (47.5)	691 (52.5)	4.59 (0.205)
	Occasional	1039 (36.6)	477 (45.9)	562 (54.1)	
	Moderate	414 (14.6)	175 (42.3)	239 (57.7)	
	Severe	68 (2.4)	27 (39.7)	41 (60.3)	

* Adjusted standardized residuals > 1.96; ** Adjusted standardized residuals < −1.96; χ^2^ = chi-squared; *p* = significance.

**Table 2:** Differences as a function of educational stage (1st–2nd, 3rd–4th grades of CSE and Post-secondary Education) in the risks (*n* = 3212, except for the case of cyber dating abuse with *n* = 1061).

	1st–2nd Grade of CSE ^a^ (*n* = 1714)		3rd–4th Grade of CSE ^b^ (*n* = 1307)		Post-Secondary Education ^c^ (*n* = 191)		*F*	*p*	η^2^	Post Hoc (Games-Howell)
	*M*	*SD*	*M*	*SD*	*M*	*SD*				
Cyberbullying victimization	1.64	3.42	2.10	3.39	1.81	2.90	6.29	0.002	0.004	a < b
Cyber dating abuse victimization	0.97	2.47	1.00	2.39	1.26	3.66	0.26	0.774	0.001	
Online grooming	0.51	2.17	1.06	3.04	1.04	2.60	16.92	0.001	0.011	a < b, c
Sexting	0.14	0.83	0.29	1.08	0.50	1.25	14.27	0.001	0.011	a < b < c
Problematic Internet use	16.37	14.88	20.96	14.29	21.11	12.94	35.84	0.001	0.024	a < b, c

Note: M = arithmetic mean; SD = standard deviation, *F* = Welch’s-*F*, *p* = significance; η^2^ = eta squared.

**Table 3:** Total correlations between the risks of the study in boys and girls.

	1.	2.	3.	4.	5.	*n*	*M* (*SD*)
Cyberbullying victimization	—	0.294	0.308	0.201	0.325	3067	1.84 (3.39)
Cyber dating victimization	0.370	—	0.310	0.155	0.201	1061	1.01 (2.67)
Online Grooming	0.424	0.255	—	0.437	0.273	3131	0.77 (2.60)
Sexting	0.339	0.234	0.640	—	0.170	3120	0.22 (0.98)
Problematic Internet use	0.251	0.247	0.142	0.150	—	2837	18.56 (14.70)

Note: The correlations for boys are shown below the diagonal and for girls above it. All correlations are significant at *p* < 0.001. *M* = arithmetic mean; *SD* = standard deviation.

**Table 4:** Comorbidity between the different Internet risks of participants susceptible to presenting all the risks.

	One Internet Risk				Two Internet Risks						Three Internet Risks				All the Risks	No Problem
	CB	CD	Grom	Sext	CB & CD	CB & Grom	CB & Sext	CD & Grom	CD & Sext	Grom & Sext	CB & CD & Grom	CB & CD & Sext	CB & Sext & Grom	CD & Sext & Grom	CB & CD & Sext & Grom	
*f*	204	31	54	33	34	85	39	8	6	26	43	18	48	8	37	435
% ^1^	18.4	2.8	4.9	3.0	3.1	7.7	3.5	0.7	0.5	2.3	3.9	1.6	4.3	0.7	3.3	39.2
% ^2^	30.3	4.6	8.0	4.9	5.0	12.6	5.8	1.2	0.9	3.9	6.4	2.6	7.1	1.2	5.5	

Note: Participants are assigned exclusively to one of categories or combination of them. All those who have any level of risk (mild, moderate or severe) are included. (^1^) Percentage of participants who completed all the items concerning risks related to victimization (*n* = 1109); (^2^) percentage over the total of those who suffer at least one risk (*n* = 674). Legend: *f* = frequency CB = cyberbullying victimization; CD = cyber dating abuse victimization; Grom = online grooming; Sext = sexting.

**Table 5:** Adjustment of comorbidity profiles 1-6 of the different Internet risks.

# Profiles	AIC	BIC	Sample Size-Adjusted BIC	LL	*p*-Value for BLRT	Entropy
1	12,941.527	12,989.673	12,957.914	6460.764	-	1
2	11,957.538	12,034.571	11,983.757	5962.769	0.001	0.993
3	11,966.113	12,072.033	12,002.164	5961.056	0.389	0.850
**4**	**10,683.626**	**10,818.433**	**10,729.509**	**5313.813**	**-**	**0.996**
5	10,695.494	10,859.188	10,751.209	5313.747	-	0.667

AIC: Aikake Information Criterion; BIC: Bayesian Information Criterion; LL: Likelihood logarithm; BLRT: bootstrapped likelihood ratio test. The selected model is in boldface.
